# The role of extracellular histones in systemic-onset juvenile idiopathic arthritis

**DOI:** 10.1186/s13052-019-0605-2

**Published:** 2019-01-14

**Authors:** Xiao Hu, Qiuling Xie, Xi Mo, Yanliang Jin

**Affiliations:** Department of Rheumatology, Shanghai Children’s Medical Center, Shanghai Jiaotong University, School of Medicine, Shanghai, 200127 China

**Keywords:** Extracellular histones, Heparin, Neutrophil extracellular traps, Systemic-onset juvenile idiopathic arthritis

## Abstract

**Background:**

To explore the effects of extracellular histones released by activated neutrophils on systemic-onset juvenile idiopathic arthritis (SoJIA), and to study the change of serum histone level between the active and remissive stage of SoJIA, then to clarify the role of serum histone in the pathogenesis of SoJIA.

**Methods:**

Twenty-six patients with SoJIA were recruited, and clinical informations were collected, and the serum histone was detected by ELISA. While neutrophils from normal children were incubated with the serum from the patients with SoJIA, also including incubated with FeCL3 and histone, the extracellular histone was detected, respectively; heparin was added to the above-mentioned groups to observe the changes of extracellular histone levels. The proportions of neutrophils, which released NETs, were calculated by confocal microscope.

**Results:**

The levels of serum histones in active SoJIA group (0.90 ± 0.90) were significantly higher than in remissive SoJIA group (0.17 ± 0.10) (*P* = 0.0009), and also higher than in control group (0.14 ± 0.09) (*P* = 0.246). Histone affects on clinical manifestations (including fever, rash, joint pain, liver and spleen enlargement, and serositis), except for joint pain. The proportions of neutrophils releasing NETs, that neutrophils were incubated with the serum from active SoJIA group, were 31.93% significantly higher than 12.32% from remissive SoJIA group (*P* < 0.0001). The proportions of neutrophils releasing NETs, that neutrophils were incubated with different concentration FeCl3 or with different concentration histones respectively, were positively correlated with the concentration of incubation; while heparins were added, NETs from neutrophils could be reduced effectively.

**Conclusions:**

The level of serum histone is positively correlated with the activity of SoJIA. Serum histone may be from NETs, which were released by activated neutrophils. Free iron can activate neutrophils to produce NETs, which may release histones, and histones can further promote NETs to be released, that results in a positive feedback loop of histones, and that may be one of the pathogenesis of acute SoJIA or MAS secondary to SoJIA. Histones maybe play one of important roles in the pathogenesis of SoJIA. Heparin can act on histones to prevent histone-induced inflammation.

**Trial registration:**

ChiCTR-OOC-15006228. Registered 9 April 2015, http://www.chictr.org.cn/showproj.aspx?proj=10752

**Electronic supplementary material:**

The online version of this article (10.1186/s13052-019-0605-2) contains supplementary material, which is available to authorized users.

## Introduction

Systemic-onset juvenile idiopathic arthritis (SoJIA) is the one of seven subtypes of juvenile idiopathic arthritis (JIA) [1]. SoJIA represents up to 10–20% of all JIA categories and is characterized by chronic arthritis, intermittently high, spiking temperatures up to 40 °C, maculopapular rash, hepatosplenomegaly, lymphadenopathy, serositis and a marked increase in the level of acute-phase reactants such as C-reactive protein (CRP) and erythrocyte sedimentation rate (ESR) [2]. SoJIA is a systemic inflammatory disease, and its etiology remains unclear.

SoJIA is obviously different from other subtypes of JIA. SoJIA has been suggested that innate immune system abnormalities seem to be more relevant to the pathogenesis of SoJIA than adaptive immune system abnormalities [3, 4]. Cimaza et al. [5] proposed that SoJIA should be classified as a self-inflammatory reaction disease rather than an autoimmune disease.

The main pathogenesis of SoJIA is phagocytic dysfunction, also including neutrophils. Therefore, the activity of SoJIA may be also related to the activation of neutrophils [6]. Other recent findings show that the neutrophil extracellular traps (NETs) may be released to outside of activated neutrophils, and NETs contain large quantities of histones, termed as extracellular histone,which are involved in inflammation and play a key role in the development of many diseases [7–10]. So, this study first focuses on the role of extracellular histone in the pathogenesis of SoJIA.

## Methods

### Patients and samples

In this study, patients with SoJIA were recruited from 2015 to 2016 at the Department of Rheumatology, Shanghai Children’s Medical Center (Shanghai, China). All patients were diagnosed with SoJIA according to the criteria of the International League of Rheumatology Alliance (ILAR). The remissive status of SoJIA was defined by the Wallace criteria, including: ①No joints with active arthritis, ②No fever, rash, serositis, splenomegaly, or generalized lymphadenopathy attributable to JIA, ③No active uveitis (to be defined), ④Normal ESR or CRP, ⑤Physician’s global assessment of disease activity indicates no disease activity for a minimum of 6 continuous months. There were 12 paired blood samples from 12 SoJIA patients in the active and remissive stages, respectively, and there were 14 blood samples from 14 SoJIA patients in active stages (for these children, after the disease were diagnosed and got preliminary control, they back to the local hospital for further treatment). All 26 cases were with complete clinical datum. 30 healthy children were used as control group. All patients gave written consent to this study, which was approved by the hospital Ethical Review Board. The clinical trial registration number is ChiCTR-OOC-15006228.

### Isolate serum

Whole blood with EDTA as anticoagulant, Centrifuge for 15 min at 300 x g, transfer upper layer fresh tubes, Centrifuge for 5 min at 1500 x g. Supernatants were collected into 1.5 ml Eppendorf tubes and stored at − 80 °C until use.

### Histone ELISA

Serum histone levels were measured by Cell Death Detection ELISA kits (Roche) according to the manufacturer’s instructions. Briefly, reconstitute the Anti-Histone Biotin and Anti-DNA POD in 450 μl double distilled water and dissolve ABTS tablets in 15 ml Substrate Buffer. The Immunoreagent is prepared by mixing of 1/20 volume Anti-DNA-POD and 1/20 volume Anti-histone-biotin with 18/20 volumes Incubation Buffer. Add to each well 20 μl serum sample and 80 μl of the Immunoreagent. Incubate on a MP shaker under gently shaking (300 rpm) for 2 h at room tempreture. Remove the solution thoroughly and rinse each well 3× with 250 μl Incubation Buffer. Pipette to each well 100 μl ABTS solution. Incubate on a plate shaker at 250 rpm for 15 min. Pipette to each well 100 μl ABTS Stop Solution. Measure and analyze at 405 nm against 490 nm wavelength.

### Neutrophil isolation

For neutrophil purification, blood was collected from healthy individuals separation by centrifugation on Histopaque-1119 (Sigma-Aldrich). The neutrophil-rich phase was collected, washed with PBS, and separated from erythrocytes on a 65–85% Percoll gradient (GE Healthcare). Neutrophils were collected from the 70–75% layer, washed with PBS, and resuspended in RPMI-1640 (GIBCOL). Neutrophils seeded in the 24-well plates were allowed to settle for 1 h at 37 °C under 5% CO_2_ prior to further experiments. Purity of neutrophils was determined with a CyFlow Space flow cytometer.

### NETs generation assay

Neutrophils seeded in the 24-well plates were allowed to settle for 1 h at 37 °C under 5% CO_2_. Isolated neutrophils were stimulated with serum from patients or healthy controls, FeCl_3_(1 μg/ml、2 μg/ml、5 μg/ml) and calf thymus histones(10 μg/ml、20 μg/ml、50 μg/ml) within or without heparin(50 μg/ml) for 4 h. Then fixed with 4% paraformaldehyde dissolved in PBS at least 1 h. The fixed cells were then stained with PI (Invitrogen) for 15 min, washed with PBS for three times, and examined under a confocal microscope (Laica). NETs generation from various treated cells was quantified by counted in a minimum of 20 fields, and they were expressed as percentage of the total number of cells. All of the results were validated at least 3 times.

### MPO ELISA

MPO levels from cell supernatant were measured by Human Myeloperoxidase Quantikine ELISA Kit(R&D)according to the manufacturer’s instructions. Add 50 μl sample and 100 μl Assay Diluent to each well. Cover with a plate sealer, and incubate at room temperature for 2 h on a horizontal orbital microplate shaker. Aspirate each well and wash× 4. Add 200 μl of Conjugate to each well, and incubate at room temperature for 2 h on the shaker. Aspirate each well and wash× 4. Add 200 μl Substrate Solution to each well, and incubate at room temperature for 20 min, protect from light. Add 50 μL of Stop Solution to each well. Read at 450 nm within 30 min, and set wavelength correction to 540 nm.

### Statistical analysis

Data were analyzed by SPSS statistics 20.0 software. Descriptive statistics for continuous parameters are presented as mean ± SD, T test was used for comparisons between two groups. Statistical significance in multiple comparisons was by one-way analysis of variance (ANOVA). Categorical variables were expressed as percentages. And were calculated with Fisher’s Exact Test. *p* < 0.05 was considered statistically significant.

## Results

### Characteristics of the children with SoJIA

A total of 26 patients with SoJIA (18 male and 8 female) were enrolled in the study, and their mean age was 8.88 ± 2.69 years (age range, 3–13 years). There were 13 girls and 17 boys in control group, and their mean age was 7.50 ± 3.00 years (age range, 3–13 years). There are no significantly differences in sex-matched between two groups (*P* = 0.412). There is no significantly difference in age between two groups (*P* = 0.5777), which are shown in Additional file 1: Table S1.

The detailed clinical manifestations and treatment schemes for the 26 patients are shown in Table 1.

**Table 1:** Characteristics of the children with SoJIA.

Subject	
Patients (n)	26
Sex (M/F,n)	18/8
Age (x ± s)	8.88 ± 2.69
Clinical manifestations	
Fever [n(%)]	14(53.8%)
Rash [n(%)]	11(42.3%)
Joint pain [n(%)]	13(50.0%)
Hepatosplenomegaly and serositis [n(%)]	12(46.2%)
Treatment schemes	
Glucocorticoid [n(%)] DMARDs	26(100%)
MTX [n(%)]	14(53.8%)
CsA[n(%)]	8(30.8%)
Others [n(%)]	2(7.7%)
Biologics [n(%)]	2(7.7%)

### 2. Serum histone level in the active and remissive SoJIA

**Fig. 1:**
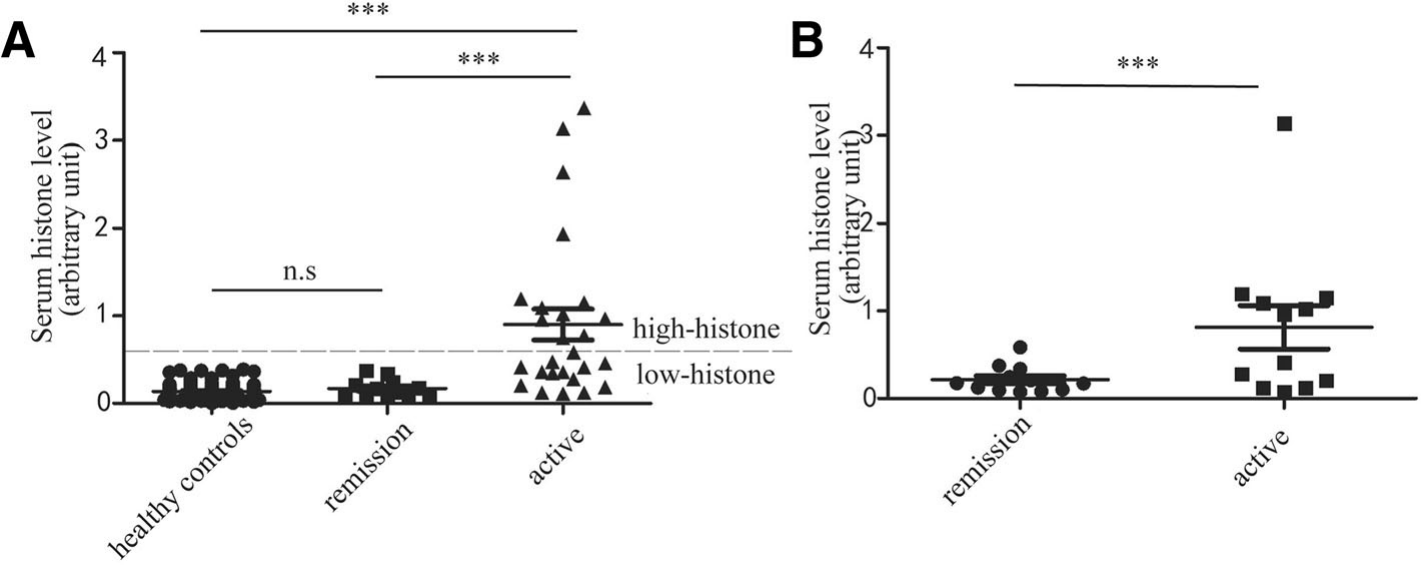
Serum histone levels during the active and remission stages of SoJIA. **a** histone levels in healthy controls, the active and remission stages of SoJIA patients’ serum; **b** the serum histone levels in paired active and remission samples from 12 children with SoJIA。*** *p* < 0.001, ** *P* < 0.01, * *p* < 0.05, n.s *p* > 0.05

As shown in Fig. 1a, the serum histone level in active SoJIA group (active group) (0.90 ± 0.90) was markedly higher than remissive SoJIA group (remissive group) (0.17 ± 0.10, *p* = 0.0009) and control group (0.14 ± 0.09, *p* < 0.0001). There was no significantly difference in serum histone level between in remissive group and in control group (*p* = 0.246). The serum histone level in paired samples (the active and remissive stage of SoJIA from the same patient) from 12 patients with SoJIA indicated that the serum histone level in active group (0.81 ± 0.86) was much higher than in remissive group (0.17 ± 0.10, *p* = 0.016). based on paired t-tests statistical method, that indicates that the serum histone level may be positively correlated with the activity of SoJIA.

### 3. Clinical manifestations between the low and high serum histone level groups

As indicated above, the serum histone level may be positively correlated with the activity of SoJIA. It was analyzed that the effect of histone levels on the activity of SoJIA according to the clinical symptoms. Based on the currently accepted guideline, to distinguish between “low-histone” and “high-histone” group, a cutoff was set above 3 SDs of the mean value for the healthy controls [11]; then, the patients were divided into the high-histone group (> 0.42) and low-histone group (< 0.42). Thereafter, clinical manifestations (including fever, rash, joint pain, liver and spleen enlargement, and serositis) were analyzed between high-histone group and low-histone group; except for joint pain, all the other clinical symptoms were significantly different between the two groups (*p* < 0.05) (Table 2), that suggested that histone affect on these clinical manifestations, except for joint pain.

**Table 2:** Clinical manifestations among the groups with low and high serum histone levels

Clinical manifestations	Low-histone(11)	High-histone(15)	*p*
Fever [n(%)]	1(9.1%)	13(86.7%)	< 0.001
Rash [n(%)]	1(9.1%)	10(66.7%)	0.005
Joint pain [n(%)]	4(36.4%)	9(60.0%)	0.428
Hepatosplenomegaly and serositis [n(%)]	2(18.2%)	10(66.7%)	0.021

### 4. Stronger ability of serum from active group to promote neutrophils to release NETs

**Fig. 2:**
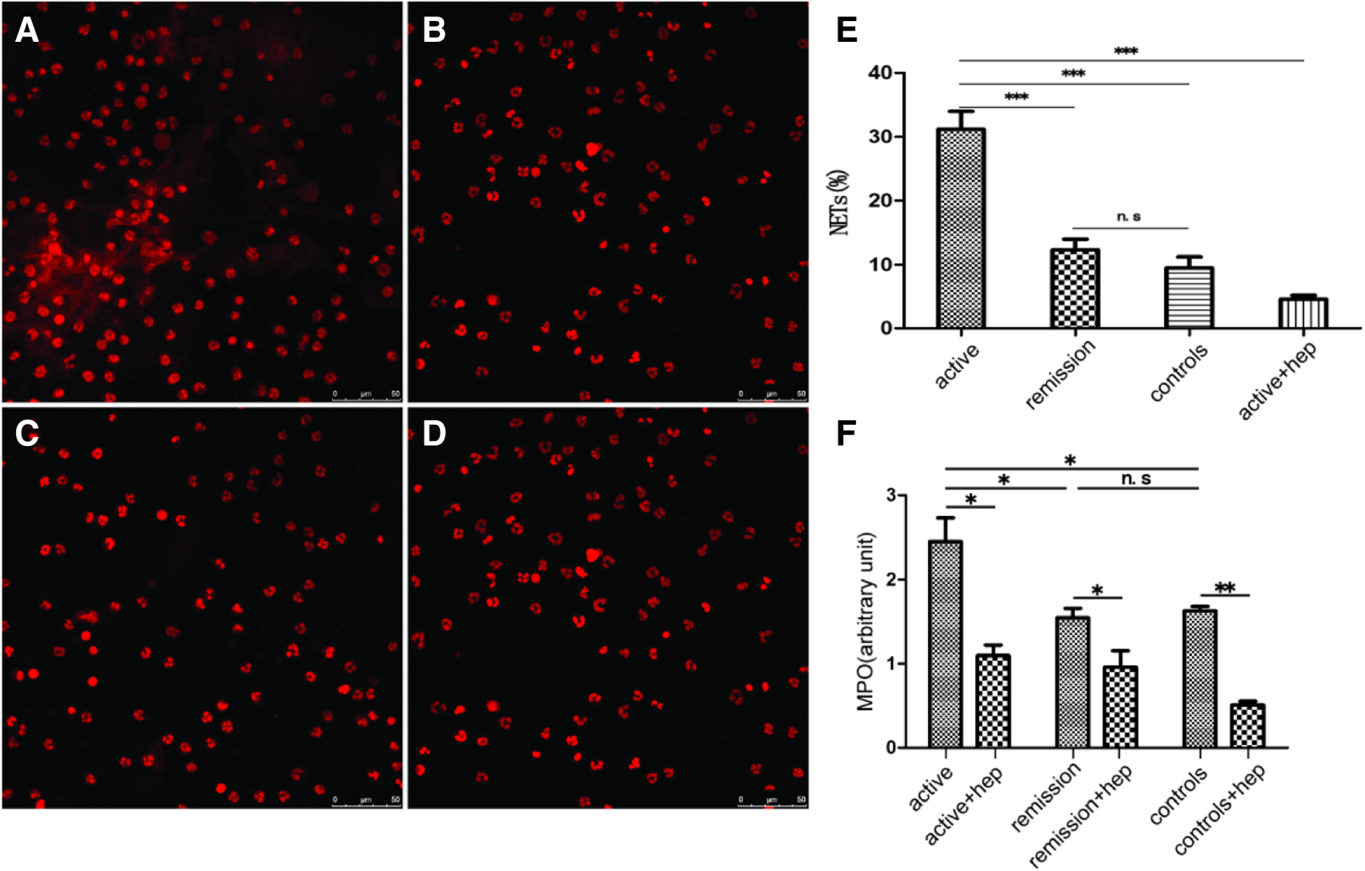
The ability of serum-stimulated NETs secreted by neutrophils, and heparin reduces active SoJIA serum-induced NETs. Serum from **a** active patients, **b** remission patients, **c** healthy controls and **d** active patients+heparin incubate with neutrophils for 4 h; E. The percentages of neutrophils releasing NETs when treated with serum from active patients, remission patients or healthy controls incubate with neutrophils for 4 h; F. the supernatant MPO levels in the active SoJIA, remission, and control groups.*** *p* < 0.001;** *P* < 0.01;* *p* < 0.05;n.s *p* > 0.05.

The serum from patients with active and remissive SoJIA was incubated with neutrophils from healthy children, and the serum from healthy children was used as a control group. The results were that the percentages of neutrophils releasing NETs were 31.93, 12.32 and 11.31%, respectively, while treated with serum from active SoJIA, remissive SoJIA and control group; Chi-square tests indicated that the percentages of neutrophils, which produced NETs, was significantly higher in the active group than in remissive and control group (*p* < 0.0001), whereas there were no significant difference between remissive and control group (*p* = 0.230). Considering that MPO is a marker of neutrophil activation, the level of extracellular MPO can objectively reflect the activation degree of neutrophils [12]. Therefore, while detecting NETs, MPO in supernatants also be detected to make sure whether neutrophils were activated and to release NETs in the above-mentioned experimental groups. The MPO levels were (2.45 ± 0.49) in active group, and (1.63 ± 0.08) in remissive group, and (1.54 ± 0.19) in control group, respectively. The MPO levels were significantly higher in the active group than in the remissive group and control group (*p* < 0.05), whereas the MPO levels did not significantly differ between the remissive group and control group (*p* = 0.5331). This result suggests that neutrophils can be actived by the serum from active SoJIA, then, NETs can be released by actived neutrophils (Fig. 2).

### Free ferric ions induce neutrophils to produce NETs

In this study, neutrophils from healthy children were incubated with different concentrations of FeCl_3_ for 4 h, and the same amount of ddH_2_O was added to the another group (neutrophils also from healthy children) as a control. Then, the proportion of neutrophils, which produced NETs, was analyzed by confocal microscope. The results were that while neutrophils from healthy children were incubated with different concentrations of FeCl_3_ (1 μg/ml, 2 μg/ml, and 5 μg/ml FeCl_3_) for 4 h, the proportion of neutrophils, which produced NETs, was 24.70, 33.33, and 56.28%, respectively, and the proportion of neutrophils, which produced NETs was only 6.71% in control group; there were significant differences among them (*p* < 0.05); the MPO level in supernatant from the the above experimental group were (1.39 ± 0.17), (1.71 ± 0.08), (1.96 ± 0.07) (in different concentrations of FeCl_3_ groups), and (0.71 ± 0.06) (in control group), respectively; there were significant differences among groups (*P* < 0.05). These results suggest that free ferric ions can activate neutrophils to produce NETs, that is a positively correlated with the concentration of FeCl_3_.

### Serum histone induces neutrophils to release NETs

Histone is also a pro-inflammation. To clarify whether histone released by activated neutrophils can stimulate neutrophils to produce NETs again, that aggravates inflammation. The neutrophils from healthy children were incubated with different concentrations of histone protein level (10 μg/ml, 20 μg/ml, and 50 μg/ml histone group) for 4 h, and then to observe these neutrophils under the confocal microscope, that the proportion of neutrophils, which produced NETs, were calculated; the same amount of ddH_2_O was added to the another group (neutrophils from healthy children) as a control. The proportion of neutrophils, which produced NETs, was 32.02, 32.51, and 45.96% in different concentrations of histone protein level (10 μg/ml, 20 μg/ml, and 50 μg/ml histone group), respectively, and the proportion of neutrophils, which produced NETs was only 6.71% in control group. There were significant difference between 50 μg/ml and 10 μg/ml groups, or 50 μg/ml and 20 μg/ml groups (*P* < 0.05), except for the 10 μg/ml and 20 μg/ml histone groups (*P* = 0.9351). Since there was no significant difference in the proportion of neutrophils producing NETs between the 10 μg/ml and 20 μg/ml histone group, the nuclear morphology of neutrophils was further analyzed, as reported by Chowdhury et al. [12]. Chowdhury et al. [12]] defined neutrophils as lobulated neutrophils (no NETs), diffused neutrophils (the produced NETs are restricted to the region around the nucleus), and spread neutrophils (the produced NETs are not limited to the region around the nucleus), according to the ability of neutrophils to produce NETs; the spread type is associated with the release of a greater amount of NETs. Therefore, the nuclear morphology of neutrophils were analyzed between 10 μg/ml and 20 μg/ml histone groups. In detail, the proportions of diffused and spread neutrophils in the 10 μg/ml histone group were 14.04 and 16.67%, and in the 20 μg/ml histone group were 11.30 and 22.60%, respectively. It suggests that although there is no significant difference in the proportion of neutrophils producing NETs between the 10 μg/ml and 20 μg/ml histone group, there is significant difference in the proportion of spread neutrophils (*P* < 0.001), that suggests that the ability of neutrophils to produce NETs in 20 μg/ml histone group is stronger than in 10 μg/ml. So the results indicate that histone could induce neutrophils to release NETs by a dose-dependent manner.

The MPO level in the supernatant from the different concentrations of histone group (10 μg/ml, 20 μg/ml, and 50 μg/ml) were (1.40 ± 0.11), (1.70 ± 0.03), and (2.03 ± 0.15), and was (0.71 ± 0.06) in the control group, respectively, that were significant differences among groups (*P* < 0.05) (Fig. 4).

### Heparin can reduce NETs released by neutrophils activated by histone or free iron

Recent studies indicated that heparin could neutralize histone toxicity [13–15]. To study the effect of heparin on neutrophil activation, heparin (50μg/ml) was added to the neutrophils incubated with different concentrations of histones or FeCl3 for 4 h, respectively, and the proportion of neutrophils producing NETs also was calculated, respectively, and no heparin was used as a control group. The proportions of neutrophils producing NETs in heparin group versus in control group (different concentrations of histones10μg/ml, 20 μg/ml, and 50 μg/ml, respectively) were 2.04% versus 32.02% (*P* < 0.0001), and 4.55% versus 32.51% (*P* < 0.0001), and 9.80% versus 45.96% (*P* < 0.0001), respectively, and there were significant differences among groups. In addition, MPO in supernatants also be detected to make sure that whether the activation of neutrophils can be inhibited through heparin neutralizing histone, and to reduce the release of NETs by activated neutrophils. MPO level in the supernatant from the different concentrations of histone group (10 μg/ml, 20 μg/ml, and50μg/ml), which were added to heparin versus control group, were (0.63 ± 0.05) versus (0.71 ± 0.60) (*P* < 0.05), and (0.73 ± 0.17) versus (1.40 ± 0.11) (*P* < 0.05), and (0.87 ± 0.06) versus (1.70 ± 0.03) (*p* < 0.05), respectively; there were significant differences among groups.

Similarly, while heparin (50μg/ml) was added in the neutrophils incubated with different concentrations of FeCl3 (1 μg/ml, 2 μg/ml, and 5 μg/ml) for 4 h, the proportions of neutrophils producing NETs in heparin groups versus no heparin groups(as control group), respectively, were 7.08% versus 24.70% (*P* < 0.05), and 5.55% versus 33.33% (*P* < 0.05), and 4.24% versus 56.28% (*P* < 0.05), respectively; there were significant differences among groups. The MPO level in the supernatant from the different concentrations of FeCl_3_ group (1 μg/ml, 2 μg/ml, and 5 μg/ml), which were added to heparin (50μg/ml) versus control group, were (0.68 ± 0.17) versus (1.39 ± 0.17) (*P* < 0.05), and (0.81 ± 0.14) versus (1.71 ± 0.08) (*P* < 0.05), and (0.94 ± 0.04) versus (1.96 ± 0.07) (*P* < 0.05), respectively; there were significant differences among groups.

**Fig. 3:**
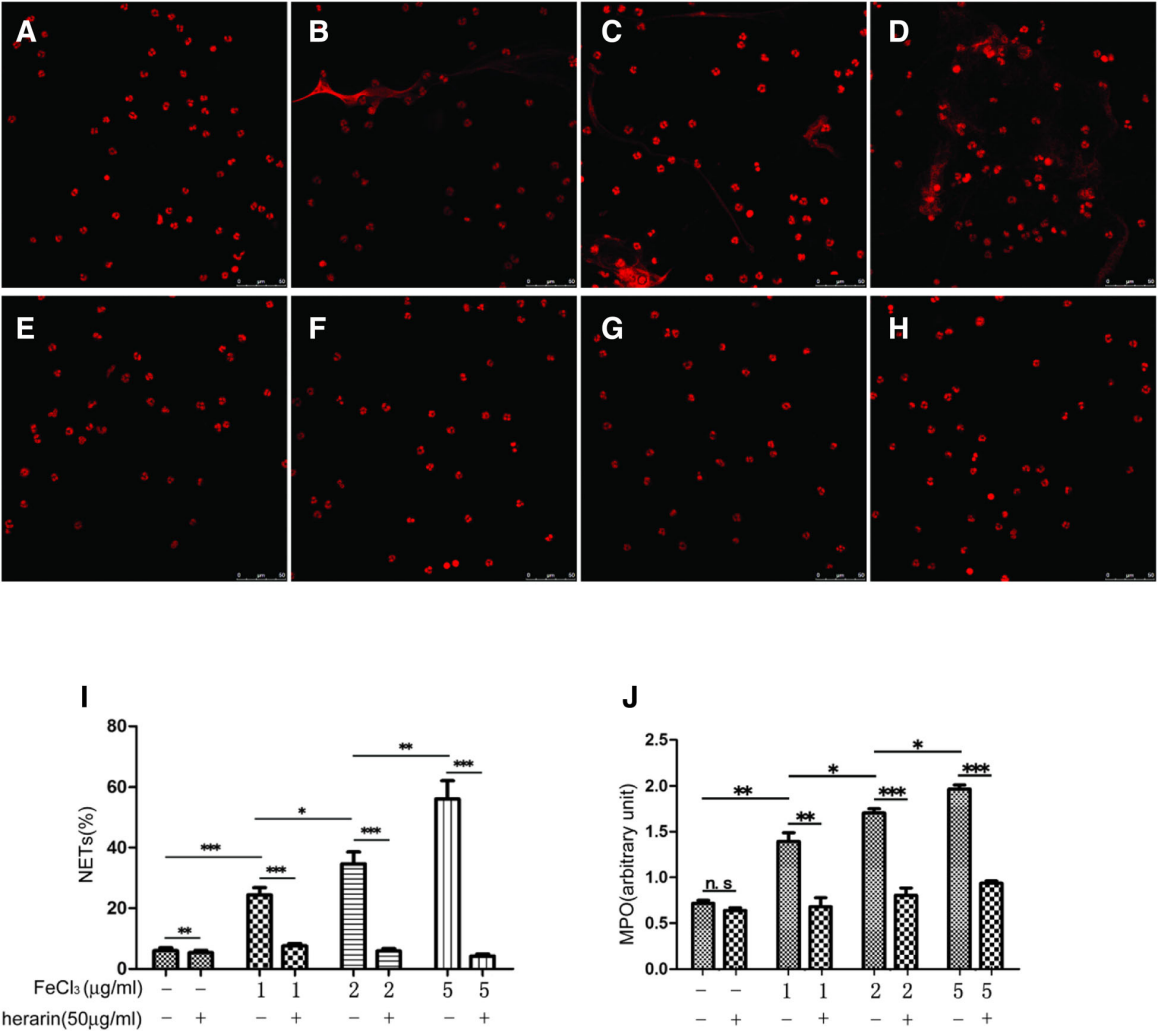
Free ferric ions induce neutrophils to produce NETs, and heparin reduces free iron-induced NET production. A: 0 μg/ml FeCl_3_, B. 1 μg/ml FeCl_3_, C. 2 μg/ml FeCl_3_, D. 5 μg/ml FeCl_3_, E. 0 μg/ml FeCl_3_ + heparin, F. 1 μg/ml FeCl_3_ + heprin, G. 2 μg/ml FeCl_3_ + heparin, H. 5 μg/ml FeCl_3_ + heprin incubate with neutrophils for 4 h; I. the proportion of neutrophils that produced NETs, and J. the supernatant MPO levels when 0 μg/ml、1 μg/ml、2μg/ml and 5 μg/ml FeCl_3_ + heparin incubate with neutrophils for 4 h. *** *p* < 0.001;** *P* < 0.01;* *p* < 0.05;n.s *p* > 0.05e

**Fig. 4:**
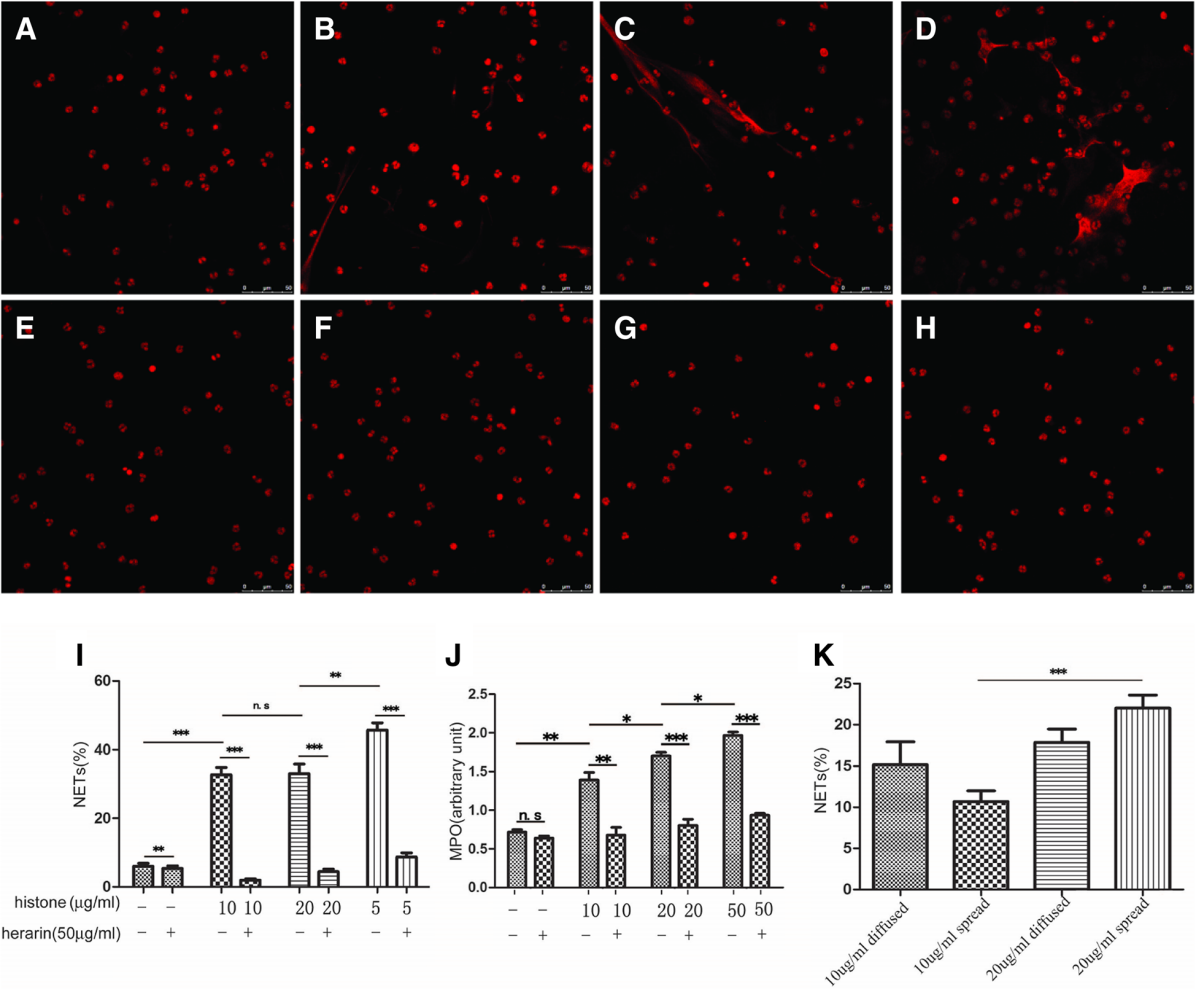
Serum histone induces neutrophils to release NETs,heparin reduces histone -induced NET production. A: 0 μg/ml histone, B. 10 μg/ml histone, C. 20 μg/ml histone, D. 50 μg/ml histone, E. 0 μg/ml histone, F. 10 μg/ml histone+heparin, G. 20 μg/ml histone+heparin, H. 50 μg/ml histone+heparin, incubate with neutrophils for 4 h; I. the proportion of neutrophils that produced NETs, and I. the MPO levels in the supernatant when neutrophils incubate with 0 μg/ml, 10 μg/ml, 20 μg/ml and 50 μg/ml histone +heparin; K. the proportions of diffused and spread neutrophils in the 10 μg/ml and 20 μg/ml histone . *** *p* < 0.001;** *P* < 0.01;* *p* < 0.05;n.s *p* > 0.05

These results suggest that heparin can effectively inhibit the activation of neutrophils by histone or FeCl3, then to reduce the release of NETs by activated neutrophils. (Figs. 3 and 4).

### Heparin can reduce the release of NETs induced by active SoJIA serum

Heparin (50 μg/ml) was added in the neutrophils incubated with serum from patient with active SoJIA. and the results were that the proportions of neutrophils producing NETs was 4.73% in heparin group and 31.93% in no heparin group, respectively, that there were significant differences among groups (*P* < 0.0001); MPO level from supernatant in heparin group and in no heparin group were (1.09 ± 0.22) and (2.45 ± 0.49), respectively, that there were significant differences between groups (*P* = 0.01). The results indicate that heparin can reduce the release of NETs induced by the serum from active SoJIA. (Fig. 2).

## Discussion

In this study, it focuses on that whether neutrophils are activated in active SoJIA, and whether the histone released by activated neutrophils may aggravate SoJIA. Then, it was found that the serum histone level in active SoJIA group (active group) was significantly higher than in remissive SoJIA group (remissive group), that suggests that the serum histone level is positively correlated with the activity of SoJIA.

Recent studies [16] have found that extracellular histones in serum are mainly derived from NETs produced by activated neutrophils. Therefore, in this study, neutrophils incubated with serum, which were from three groups (including active group, remissive group, and control group). The proportion of neutrophils producing NETs in the active group was the highest among three groups, that suggested that the serum histones in patients with active SoJIA are produced by activated neutrophils. Moreover, the affect of serum histone on clinical manifestations (including fever, rash, joint pain, hepatosplenomegaly, and serositis) were also discussed in SoJIA. The patients were divided into two groups, which include low histone level group and high histone level group according to the currently accepted guidelines [11]. It was found that except for joint pain, the frequency of all the other clinical symptoms (including fever, rash, hepatosplenomegaly, and serositis) was significantly higher in the high histone group than in low histone group. This result demonstrates that serum histone levels are related to these clinical signs and symptoms in SoJIA.

Grom AA et al. [17] had reported that the macrophage expressing CD163 in active SoJIA can activate the oxidative stress response through pathways involving heme and free iron; eventually, hemoglobin is converted to bilirubin, CO(carbon monoxide), and free iron. The free iron is either sequestered by ferritin or transported and distributed to red blood cell precursors in the bone marrow [18]. So, these free ions require a large amount of serum ferritin for being transported, which could be one of the reasons for the high level of serum ferritin in children with active SoJIA [16, 17]. Free ions are strong oxidants which can promote inflammation [3], so free ions maybe can activate neutrophils. In this study, neutrophils from the normal children were incubated with different concentrations of FeCl3, and the MPO levels in supernatant were detected. The results confirmed that the trivalent ferric ions can induce NETs produced by active neutrophils with a dose-dependent manner. So, the serum free ions in active SoJIA may induce NETs produced by active neutrophils and lead to increased serum histone level. In order to further explore the role of histone as pro-inflammation in promoting the release of NETs by neutrophils, the neutrophils were incubated with different concentrations of histone. It was found that the effect of histones on NETs released by neutrophils was generally dose-dependent. Interestingly, there was no significant difference in the proportion of neutrophils producing NETs between the 10 μg/ml and 20 μg/ml histone groups. Then, nuclear morphology of neutrophils were analyzed between two groups according to Chowdhury et al. reported [12], and there is significant difference in the proportion of spread neutrophils (*p* < 0.001), that suggests that the ability of neutrophils producing NETs in 20 μg/ml histone group is stronger than in 10 μg/ml. So, it can be inferred that while SoJIA is active, or macrophage activation syndrome (MAS) is occurring, the macrophages increase expressing CD163 [19, 20], that maybe lead to the elevation of serum free iron levels, then to activate neutrophils to release NETs, that lead to increase histone level in serum. Thereafter, the elevated histone level can further activate the neutrophils to release NETs, which can then induce histone release once more, thus to form a vicious positive feedback mechanism for increasing inflammation and exacerbating disease progression. Extracellular histone level may be a risk/injury-related molecular pattern and reflect the extent of organ and tissue damage [16]. In fact, extracellular histones have been found to cause damage to the lung, brain, liver, kidney, and other organs [21–24]. By killing other cells and activating TLRs, NLRP3, and other inflammation, histone aggravates tissue damage. Now, histone is therefore considered to be a potential therapeutic target for the treatment and control of inflammatory diseases.

Heparin has been found to be able to neutralize the toxicity of histone in vitro and in vivo [13–15]. In the treatment of sepsis, it is proposed that heparin can act on histone to prevent histone-induced inflammation through breaking the vicious cycle, then to avoid further damage to organs [16]. In this study, it was also confirmed that heparin can effectively prevent the release of NET caused by active neutrophils, which were activated by active SoJIA serum, histone, and FeCl3. It is not clear that how heparin affects on histone, one of the mechanisms proposed maybe involves the potential electrostatic adsorption between histones and heparin molecules, which leads to their interactions [13, 25].

In this study, it is found that NETs released by activative neutrophils, and then a large number of histones were released from NETs, that leads to form a malignant positive feedback mechanism of inflammatory damage. This may be one of the important factors that aggravates SoJIA, So, histone may become one of the potential targets for the treatment of SoJIA, that will be helpful to provide new ideas and strategies for the study of pathogenesis and treatment of SoJIA.

## Conclusions

The level of serum histone is positively correlated with the activity of SoJIA. Serum histone may be from NETs released by activated neutrophils. Free irons can activate neutrophils to produce NETs, which may release histones, then histones can further promote NETs to be released, that results in a positive feedback loop of histone; the positive feedback loop of histone may be one of the pathogenesis of acute SoJIA or MAS secondary to SoJIA. So histone maybe play one of important roles in the pathogenesis of SoJIA. Heparin can act on histone to prevent histone-induced inflammation.

## Additional file

Additional file 1: **Table S1.** Age and sex in control group and Children with SoJIA (DOCX 16 kb)
