# Supplementary material for: The role of extracellular histones in systemic-onset juvenile idiopathic arthritis
Source: Ital J Pediatr. 2019 Jan 14;45:14. doi: 10.1186/s13052-019-0605-2 (PMC6332604; doi:10.1186/s13052-019-0605-2)
Supplement: Supplementary file 1 — Table S1. Age and sex in control group and Children with SoJIA (DOCX 16 kb) [file 13052_2019_605_MOESM1_ESM.docx]

Additional file 1: Table S1 Age and sex in control group and Children with SoJIA

|  | Control group | Children with SoJIA | p |
| --- | --- | --- | --- |
| Age (x±s) | 7.50±3.00 | 8.88±2.69 | 0.5777 |
| Sex (M/F,n) | 17/13 | 18/8 | 0.412. |
